# Sherlock Holmes Doesn’t Play Dice: The Mathematics of Uncertain Reasoning When Something May Happen, That You Are Not Even Able to Figure Out

**DOI:** 10.3390/e27090931

**Published:** 2025-09-04

**Authors:** Guido Fioretti

**Affiliations:** Management Department, University of Bologna, 40126 Bologna, Italy; guido.fioretti@unibo.it

**Keywords:** Evidence Theory, Belief Functions, Dempster–Shafer Theory, radical uncertainty, Constraint Satisfaction Networks, prompt engineering

## Abstract

While Evidence Theory (also known as Dempster–Shafer Theory, or Belief Functions Theory) is being increasingly used in data fusion, its potentialities in the Social and Life Sciences are often obscured by lack of awareness of its distinctive features. In particular, with this paper I stress that an extended version of Evidence Theory can express the uncertainty deriving from the fear that events may materialize, that one is not even able to figure out. By contrast, Probability Theory must limit itself to the possibilities that a decision-maker is currently envisaging. I compare this extended version of Evidence Theory to cutting-edge extensions of Probability Theory, such as imprecise and sub-additive probabilities, as well as unconventional versions of Information Theory that are employed in data fusion and transmission of cultural information. A possible application to creative usage of Large Language Models is outlined, and further extensions to multi-agent interactions are outlined.

## 1. Introduction

Sometimes, unexpected and novel events upset the network of causal relations on which we base our decisions. A global pandemic in the XXI century, a local conflict that might degenerate into World War III, as well as the 2008 financial crisis are global and well-known examples of destructive events that nobody had conceived before they actually happened. One consequence of such events is that they may suggest a simple but fundamental question: “Now, what else?”.

Such questions do not follow automatically from empirical evidence, but rather depend on existing mental models and whether we allow them to be questioned by the available evidence. Dr. Watson typically sticks to the most obvious interpretation of facts, whereas Sherlock Holmes allows apparently negligible cues to upset the received wisdom. Does Sherlock Holmes know from the very first page who is guilty? I assume not. But he knows that, whatever the truth, it will be different from anything he can currently figure out. Therefore, he starts searching for an alterative explanation.

Probability Theory (PT) cannot express the doubt that novel and potentially disruptive possibilities may materialize, precisely because one of its assumptions is that an exhaustive set of possibilities is given. Nevertheless, this sort of non-probabilistc uncertainty has been hotly debated in economics because of its impact on investment decisions [1].

This sort of uncertainty, which has been variously qualified as “Keynesian,” “fundamental,” “true,” “epistemic,” “ontological” and more recently “radical” uncertainty [1,2,3,4,5,6], should be clearly distinguished from the uncertainty deriving from lack of information on given possibilities, such as the paucity of the data on which probabilities are measured. Small sample size, unfair dice and unique events pose serious problems to the assessment of reliable probabilities, but they still concern a set of known events. With a possibly awkward but dense expression, the literature traces a clear distinction between “known unknowns” (unknown probabilities of known possibilities) and “unknown unknowns” (unknown probabilities of unknown possibilities) [7,8,9].

While the problem of known unknowns is interesting in itself, I rather focus on the more challenging problem posed by unknown unknowns. Specifically, the reason for writing this essay is that Evidence Theory (ET) [10] and its subsequent improvements provide a framework to deal with unknown unknowns, first of all by rejecting the assumption that the complementation operator must be necessarily applied to the possibility set. Without complementation, the easy but illusory solution of defining an all-encompassing residual event is not available. Subsequently, precisely because the disturbing clues that stimulate Sherlock Holmes’s investigations cannot be moved under the carpet, radical uncertainty can be measured by the extent of contradictory, unexplained evidence.

The canonical setting of ET is a judge listening to testimonies or a detective looking for clues, rather than a gambler playing dice [11,12]. This is critical, because while it is natural for a gambler to reason in terms of a given set of outcomes, a detective must be open to unexpected denouements. While a substantial portion of the literature has limited itself to the purely technical aspects of ET, I stress its distinctive paradigm and its logical consequences. In particular, I aim at connecting with one another the awareness of the importance of radical uncertainty developed by social scientists with the ability of certain extended versions of ET to deal with it.

The rest of this paper is organized as follows. The ensuing section Section 2 illustrates the basics of ET with respect to both known and unknown unknowns. Contrary to most introductions to ET, I emphasize its degree of freedom concerning hypotheses formulation. Subsequently, Section 3 frames ET with respect to PT and Information Theory (IT), respectively. In particular, Section 3.1 illustrates the ability of ET to estimate known unknowns whereas Section 3.2 discusses the usefulness of ET for non-trivial problems of information transmission. Section 4 explores the possibility of using ET in interpersonal decision-making. Finally, Section 5 concludes with prospects for future developments and applications.

## 2. Radical Uncertainty Within Evidence Theory

This brief introduction to ET aims at highlighting its ability to express radical uncertainty. The ensuing account merges Shafer’s original ET [10] with insights from Smets’s Transferable Belief Model (TBM) [13]. In particular, the possibility to express radical uncertainty is a nice contribution of TBM that I shall extend insofar as it concerns its consequences for representing decision-making (see Appendix A for a detailed account of contributions and interpretations).

In ET, the possibility set is called *Frame of Discernment* (FoD) in order to stress that it represents what possibilities a decision-maker is envisaging at a certain point in time. Let Θ denote a FoD that entails possibilities $A_{1},$ $A_{2},\ldots$ $A_{N}$. Suppose that these possibilities are supported by masses of empirical evidence $m(A_{1}),$ $m(A_{2}),\ldots$ $m(A_{N})$, respectively.

Notably, possibilities $A_{i}$ are not necessarily disjoint sets. Testimonies support one another insofar as they correspond to (partially) overlapping sets, whereas they contradict one another insofar as they do not overlap. In general, ∀i,j it is possible that $A_{i}\cap A_{j}\neq \emptyset$. Formally, PT can be obtained as a special case of ET when all possibilities are singletons (see Section 3.1 for details).

Since in the FoD possibilities are represented as sets that may intersect with one another, $m\left(A_{i}\right)+m\left(A_{j}\right)$ is generally not equivalent to $m(A_{i}\cup A_{j})$. Thus, albeit normalization to 1 is possible and generally carried out, it does not imply that a given amount is distributed among mutually exclusive possibilities as in PT.

One other consequence of representing possibilities as sets instead of singletons is that while ET is able to deal with “known unknowns” by means of smaller masses *m* pretty much as PT does by assuming sub-additive probabilities, ET can also approach the problem of “known unknowns” by tuning overlaps between possibilities. Consider, for instance, the novel natural catastrophes that are being caused by climate change. These are “known unknowns” because floods and hurricanes existed even before the climate started to change, but the sample of events since climate has changed is too small for a reliable estimation of probabilities. In this case PT resorts to sub-additive probabilities, and quite similarly, ET can resort to assigning small values to masses *m*. However, ET can also assess the differences between climate change-induced catastrophes with the past ones by translating these differences into the contours of the corresponding possibilities in the FoD (see Section 3.1 for details).

Coming to “unknown unknowns,” ET comes in flavors where an FoD Θ may or may not be coupled to the complementation operator to form a σ-algebra. Henceforth, I shall assume that Θ is not a σ-algebra in order to exclude the possibility of encapsulating unknown unknowns into an all-encompassing residual event.

Radical uncertainty (due to unknown unknowns) originates from evidence that contradicts established relations of causes and effects, with novel possibilities eventually entering the FoD. In other words, once novel and unthinkable things have been observed, one expects other unknown unknowns to appear in the future. By leveraging on empirical investigations and theoretical considerations on abductive logic [14,15,16], I take the amount of conflicting evidence as an appropriate measure of radical uncertainty. In other words, the more the conflicting evidence, the stronger the current causal maps are questioned, hence the stronger the fear that they may be finally upset.

Within ET, the Transferable Belief Model (TBM) [13,17,18] assumes that conflicting evidence translates into m(∅)>0. The rationale of this assumption is that conflicting evidence, by suggesting that something may happen, that is currently not in the FoD, moves some mass *m* towards possibilities that cannot yet be defined.

Independently of radical uncertainty, ET also allows us to assign a positive mass to the FoD as a whole. This mass is neither committed to the possibilities that are being envisaged, nor to the void set which represents the fear that something else may happen. An m(Θ)>0 represents suspension of judgement, non-assigned belief that in the course of investigations the judge or detective may assign or withdraw from specific possibilities, or the void set. In the final denouement of detective stories, both m(∅) and m(Θ) finally shrink down to zero.

Let us suppose that possibilities $A_{1},$ $A_{2},\ldots$ $A_{N}$ are being envisaged, supported by masses of empirical evidence $m(A_{1}),$ $m(A_{2}),\ldots$ $m(A_{N})$, respectively. Let us suppose that a state of mind expressing the fear of unknown unknowns is there, which translates into m(∅)>0. Let us suppose that evidence is sufficiently scant to suggest restraint, which translates into m(Θ)>0. Though not essential to the theory, masses m(.) can be normalized in order to obtain that

(1) $$\sum_{i=1}^{N}\;m_{i}\left(A_{i}\right)\;+\;m_{A}\left(\emptyset \right)\;+\;m_{A}\left(\Theta \right)\;=\;1$$

Notably, since in ET possibilities $A_{i}$ are not necessarily disjoint sets, Equation (1) does not amount to distributing a given mass among distinct possibilities. This normalization adjusts the evidence supporting partially overlapping sets, net of judgement restraint represented by m(Θ) and the fear of unknown unknowns represented by m(∅).

Let us assume that evidence $A=\{m\left(A_{1}\right),$ $m(A_{2}),\ldots$ $m(A_{N_{A}}),$ $m_{A}\left(\emptyset \right),$ $m_{A}\left(\Theta \right)\}$ is available when a new body of evidence arrives (e.g., a new testimony, new cues, etc.). Let $B=\{m\left(B_{1}\right),$ $m(B_{2}),\ldots$ $m(B_{N_{B}}),$ $m_{B}\left(\emptyset \right),$ $m_{B}\left(\Theta \right)\}$ be this new body of evidence. Just as the sets entailed in one single body of evidence are not necessarily disjoint, ∀i,j it may either be $A_{i}\subseteq B_{j}$, or $A_{i}⊇B_{j}$, or $Ai\cap B_{j}\neq \emptyset$, or $A_{i}\cap B_{j}=\emptyset$.

The judge, or detective, must evaluate which items in these two bodies of evidence are coherent with one another while weighing them against contradictory items. In a closed-world assumption (no unknown unknowns, and therefore $m_{A}\left(\emptyset \right)=$ $m_{B}\left(\emptyset \right)=$ $m_{C}\left(\emptyset \right)=0$), Dempster–Shafer’s combination rule [10,19] yields the components of a new body of evidence *C* that unites *A* and *B*. Note that intersections with Θ enter the computation:

(2) $$m\left(C_{k}\right)\;=\;\frac{\sum_{X_{i}\cap Y_{j}=C_{k}}\;m_{A}\left(X_{i}\right)\;m_{B}\left(Y_{j}\right)}{1-\;\sum_{X_{i}\cap Y_{j}=\emptyset }\;m_{A}\left(X_{i}\right)\;m_{B}\left(Y_{j}\right)}$$

where $X_{i}\in \left\{A_{i}\;\forall i,\;\Theta \right\}$, $Y_{j}\in \left\{B_{j}\;\forall j,\;\Theta \right\}$, and where the $C_{k}$s are defined by all possible intersections of the $X_{i}$s with the $Y_{j}$s.

The numerator of Equation (2) measures the extent to which the two bodies of evidence support $C_{k}$, whereas the denominator measures the extent to which they are not contradictory with one another. In the simplest, 1-dimensional case [20], information is conveyed through a series of *n* testimonies of reliability *m* each, yielding a combined reliability $m^{n}$. By contrast, the combined reliability of *n* independent parallel testimonies is $1-{\left(1-m\right)}^{n}$. Thus, in Equation (2) the numerator expresses the logic of serial testimonies whereas the denominator expresses the logic of parallel testimonies.

Dempster–Shafer’s combination rule (2) can be iterated to combine any number of evidence bodies. Its outcome is independent of the order in which they are combined.

Rule (2) has been found to yield unsatisfactory results for closed-world problems that are framed in terms of mutually exclusive possibilities. Several solutions have been proposed, including alternative combination rules (see Appendix B for an illustration of alternatives). However, mutually exclusive possibilities are typical of gamblers playing games of chance, rather than judges listening to testimonies or detectives evaluating cues. Possibly, those are not the sort of decision problems to which ET should be applied [21,22].

The case of an open world where unknown unknowns are possible (either $m_{A}\left(\emptyset \right)>0$, or $m_{B}\left(\emptyset \right)>0$, or both) is substantially more complex. Conflict can neither be ignored nor redistributed. In open worlds, conflict is an indicator that mental models are failing, causal maps are not providing orientation, and some cognitive re-arrangement is in order. Novel possibilities are likely to emerge, that are currently impossible to conceive.

In an open world, TBM applies. TBM makes use of the numerator of Equation (2) while extending it to m(∅)[17,18]. The idea is that since m(∅)>0 expresses a degree of belief concerning possibilities that are currently not within Θ, it should not be redistributed among those that are. Smets’ combination rule is:

(3) $$m\left(C_{k}\right)\;=\;\sum_{X_{i}\cap Y_{j}=C_{k}}\;m_{A}\left(X_{i}\right)\;m_{B}\left(Y_{j}\right)$$

where $X_{i}\in \left\{A_{i}\;\forall i,\;\emptyset ,\;\Theta \right\}$, $Y_{j}\in \left\{B_{j}\;\forall j,\;\emptyset ,\;\Theta \right\}$ and the $C_{k}$s are defined by all possible intersections of the $X_{i}$s with the $Y_{j}$s.

The outcome of Smets’ combination rule must be normalized by means of Equation (1). Note that while normalization is optional for Equation (2), it is compulsory for Equation (3).

Let us suppose that, once all available bodies of evidence have been combined, the judge or detective formulates a hypothesis H. This hypothesis is a subset of Θ but, unlike the $A_{i}$s, it does not represent empirical evidence but rather a mental construct.

The belief that the judge or detective can reasonably attach to H is given by the amount of evidence supporting it. Assuming a body of evidence $C=\{m\left(C_{1}\right),$ $m(C_{2}),\ldots$ $m(C_{N_{C}}),$ $m_{C}\left(\emptyset \right),$ $m_{C}\left(\Theta \right)\}$, and in accord with TBM [23], the following *Belief Function* expresses the belief in H supported by *C*:

(4) $$Bel\left(H\right)\;=\left\{\begin{array}{ccc} \sum_{C_{k}\subset H}\;m\left(C_{k}\right) & \mathrm{if} & H\subset \Theta ,\;H\neq \emptyset \\ m_{C}\left(\emptyset \right) & \mathrm{if} & H\equiv \emptyset \\ m_{C}\left(\Theta \right) & \mathrm{if} & H\equiv \Theta \end{array}\right.$$

where the second and third lines are different from the formulation for closed worlds [10], which assumed Bel(∅)=0 and Bel(Θ)=1.

While belief in H is only supported by the evidence bearing specifically on H, it may be desirable to include also the evidence that partially supports it. In particular, as in TBM [23] I introduce the following *Plausibility Function*:

(5) $$Pl\left(H\right)\;=\left\{\begin{array}{ccc} \sum_{C_{k}\cap H\neq \emptyset }\;m\left(C_{k}\right) & \mathrm{if} & H\subset \Theta ,\;H\neq \emptyset \\ m_{C}\left(\emptyset \right) & \mathrm{if} & H\equiv \emptyset \\ m_{C}\left(\Theta \right) & \mathrm{if} & H\equiv \Theta \end{array}\right.$$

where the second and third lines are different from the formulation for closed worlds [10], which assumed Pl(∅)=0 and Pl(Θ)=1.

Obviously, Bel(H)≤Pl(H). With a more subjective interpretation, belief and plausibility can be interpreted as expressing degrees of necessity and degrees of possibility, respectively [24,25,26].

Note also that while the FoD is not allowed to generate novel possibilities by complementation (no residual events), the judge or detective can make use of any sort of operator in order to formulate hypotheses. For instance, one can assume that hypotheses are formulated in a subset Ω⊂Θ which is a σ-algebra [27]. Thus, ∀H≠∅ and ∀H≠Θ it is $Bel\left(H\right)+Bel\left(\bar{H}\right)\leq 1$ and $Pl\left(H\right)+Pl\left(\bar{H}\right)\geq 1$.

In general, decision-makers may formulate several alternative hypotheses, which they may wish to compare to one another given the available evidence. For instance, hypotheses $H_{1}$ and $H_{2}$ might be compared by evaluating either $Bel\left(H_{1}\right)≶Bel\left(H_{2}\right)$, or $Pl\left(H_{1}\right)≶Pl\left(H_{2}\right)$.

In general, hypotheses change with time. The hypotheses that are being entertained can either change out of some behavioral algorithm simulating human reasoning, or because of actual participation of a human being in subsequent interactions with an expert system, or they may be simply generated by subsequent iterations of either Equations (2) or (3), in which case $H_{k}\equiv C_{k}$. Or, some combination of the above cases. Note that ET does not impose any constraint on the process of hypotheses generation.

Hypotheses generation can be just as trivial as those generated by Dr. Watson, or as creative and surprising as those conceived by Sherlock Holmes. Since several experiments have established that conflicting evidence impairs decision-making on given possibilities [28,29,30,31], I submit that creative hypotheses generation is triggered when m(∅) goes beyond some threshold. Estimations of this threshold are only available for specific experiments [29], and they are likely to be moderated by factors that are still unknown. Heterogeneity certainly exists, with Sherlock Holmes characterized by a much lower threshold than Dr. Watson.

Hypothesis generation implies tightening or coarsening the FoD. While this aspect is generally neglected in the literature on ET, its initiator made a few illuminating remarks in this respect:

Like any creative act, the act of constructing a frame of discernment does not lend itself to thorough analysis. But we can pick out two considerations that influence it: (1) we want our evidence to interact in an interesting way, and (2) we do not want it to exhibit too much internal conflict.

Two items of evidence can always be said to interact, but they interact in an interesting way only if they jointly support a proposition more interesting than the propositions supported by either alone. (…) Since it depends on what we are interested in, any judgment as to whether our frame is successful in making our evidence interact in an interesting way is a subjective one. But since interesting interactions can always be destroyed by loosening relevant assumptions and thus enlarging our frame, it is clear that our desire for interesting interaction will incline us towards abridging or tightening our frame.

Our desire to avoid excessive internal conflict in our evidence will have precisely the opposite effect: it will incline us towards enlarging or loosening our frame. For internal conflict is itself a form of interaction—the most extreme form of it. And it too tends to increase as the frame is tightened, decrease as it is loosened.

(Glenn Shafer [10], Ch. XII.)

Albeit the early versions of TBM proceeded to a “pignistic transformation” to probabilities whenever m(∅)>0 [13,32], more recent developments are capable of attaching lower reliability to certain bodies of evidence [33,34,35] or discount correlations between different bodies of evidence [36,37]. However, just like Sherlock Holmes looks for details that finally overthrow Dr. Watson’s interpretation, the FoD should be tightened and coarsened until the sources are either sufficiently detailed to be reliable and uncorrelated with one another, or discarded altogether [38].

ET is designed for iteratively zooming the FoD until the judge, or detective, arrives at an interesting, non-trivial representation that expresses little or no contradiction [39]. This implies repeating interactions between humans and their environment in order to make sense of conflicting evidence, rather than revising Equation (2) in order to make it steer a compromise when conflicts are too large (see Appendix A). One research direction, not yet explored, could aim at exploiting the potentialities of ET in human-machine interaction.

### Example: Creativity and Hallucinations in Large Language Models

Human beings face novel, unimagined events many times in the course of their lives, most often when they must make key decisions concerning their dearest relatives and friends, their most profound beliefs and their careers. For decision-makers on top of hierarchical organizations, the radical uncertainty generated by novel technologies and unexpected political developments has a substantial impact on strategies and investment decisions, making unknown unknowns substantial in the most important decisions they make.

However, precisely those decisions are often made without disclosing information and relevant interaction. Moreover, in the rare cases when sensitive information on strategic decision-making has become available after a suitable amount of time has elapsed, that information has turned out to have been small, partial and purely qualitative.

Usage of computers in decision-making may change this state of affairs, providing opportunities to collect quantitative information while critical decisions are being made. Specifically, henceforth I shall outline a few possibilities that might be offered by Large Language Models (LLMs).

All too often, LLMs provide conventional answers. If they are set to be more creative—e.g., by tuning temperature, or other parameters [40]—they generate hallucinations along with more interesting material, i.e., answers that are clearly inconsistent with reality. In other words, hallucinations cannot be avoided if one wants the LLM to generate creative suggestions [41]. This is a man–machine interaction problem to which ET could be potentially applied.

Consider the following prompt:

What happens to you if you eat passion fruit seeds?

The simple answer is: “Nothing,” and this is what you obtain from your LLM if you know what you want. However, if one is looking for more interesting answers, one may obtain (freely adapted from [42]):

*A*_1_:  Nothing happens.
*A*_2_:  You will not digest the seeds.
*A*_3_:  The seeds will be excreted.
*A*_4_:  You will feel very happy.
*A*_5_:  You will be visited by the ghost of your dead lover.

Answers $A_{2}$ and $A_{3}$ are clearly true, and more detailed that $A_{1}$. Answer $A_{5}$ is clearly a hallucination, but you may doubt whether $A_{4}$ is a hallucination or a valid answer, at least for some varieties. If you prompted your LLM because you were looking for stimulating answers, $A_{5}$ is likely irrelevant but you accept to receive it because $A_{2}$, $A_{3}$ and $A_{4}$ are more interesting than $A_{1}$. In particular, $A_{4}$ may be a surprising possibility that you had not been able to imagine from the outset.

Suppose that you are able to assess to what extent these answers overlap, as well as how factual and reliable these answers are, which you translate into numbers $m(A_{i})$ and $m_{A}\left(\Theta \right)$. You may also assess that $A_{1}\cap A_{4}=\emptyset$ and $A_{1}\cap A_{5}=\emptyset$, that $A_{2}\cap A_{4}$ may be = or ≠∅ depending on the exact meaning your LLM attaches to the word “digest,” whereas $A_{4}\cap A_{5}$ may be = or ≠∅ depending on the sort of relation you had with your dead lover. In either case you end up with some m(∅)>0, which may prompt you to ask further questions concerning digestion and the chemical properties of passion fruits, as well as requiring your LLM to ignore ghosts. These numbers constitute a body of evidence $A=\{m\left(A_{1}\right),$ $m(A_{2}),$ $m(A_{3}),$ $m(A_{4}),$ $m(A_{5}),$ $m_{A}\left(\Theta \right)$, $m_{A}\left(\emptyset \right)\}$. In particular, the $m_{A}\left(\emptyset \right)>0$ expresses the extent to which incoming evidence is at odds with your initial expectation that “nothing happens” so you may suspect that other possibilities may come up, that you are currently unable to figure out.

Likewise, non-empty intersections between the $A_{i}$s can also suggest interesting questions. For instance, $A_{2}\cap A_{4}\neq \emptyset$ may also suggest questions about the chemical properties of passion fruits.

The analysis of the unions or intersections of possibilities $A_{i}$s may induce you to formulate hypotheses about passion fruits. For instance, a few hypotheses could be as follows:

$H_{1}$:  Passion fruits entail some psychotropic substance.
$H_{2}$:  Passion fruits boost vitamins and sugars, nothing else.
$H_{3}$:  Passion fruits have extremely hard seeds.
$H_{4}$:  There is nothing special about passion fruits, except a somewhat misleading name.

Hypotheses express what you are interested in. For instance, the above set of hypotheses could have been formulated by someone who does not care about ghosts; by contrast, LLM users with some interest in esoteric knowledge might focus on dead lovers’ apparitions. Differential focusing amounts to tightening or coarsening specific portions of the FoD.

This set of hypotheses suggests a second prompt that will generate a second body of evidence *B*, that you may combine with *A* by means of Equation (3). And so on, focusing on interesting interactions suggested by tiny details along the pattern followed by Sherlock Holmes.

Clearly, assessment of the m(.)s and the extent of $A_{i}\cap A_{j}$s is a difficult job that is still waiting to be done. However, automated text analysis may possibly help in this respect. In parrticular, it should be relatively easy to obtain reliable measures of concept overlap that express of $A_{i}\cap A_{j}$s, whereas assessment of the amount of evidence and its reliability—the numbers m(.)—is likely to pose greater practical difficulties.

## 3. Evidence, Probability, and Information Theory

This section illustrates PT and IT from the point of view of ET. Formally, ET understands PT and IT as special cases that obtain when sets $A_{i}$ are singletons—henceforth denoted as $\left\{A_{i}\right\}$—representing possibilities that can either be distinct or coincide, but never intersect. It is also necessary to assume m(∅)=0 whereas a sort of m(Θ)>0 can be contemplated by versions of PT that allow for sub-additive probabilities. With these assumptions, the mathematics of PT and IT becomes a subset of ET. However, the conceptual difference between a gambler and a judge, or detective, is bound to stay.

One common approach to ET starts from an exhaustive set of singletons $\left\{A_{i}\right\}$s whose combinations yield sets $\{A_{1},$ $A_{2},$ …} to which a “basic probability assignment” $\{m\left(A_{1}\right),$ $m(A_{2}),\ldots \}$ is assigned. For instance, $A_{5}$ could be defined as $A_{5}=$ $\{A_{27}\},$ $\{A_{4}\},$ $\left\{A_{92}\right\}$ with $m(A_{5})=0.2$. It is evident that, since the original set of singletons is given once and for all, radical uncertainty and “unknown unknowns” cannot be contemplated by this approach. I rather followed TBM which starts by assuming the existence of masses m(.) on which belief and plausibility functions can be defined [32].

### 3.1. Evidence Theory and Probability Theory

Technical and practical differences between ET and PT become apparent when Dempster–Shafer’s combination rule is compared to Bayes’ rule [43,44,45]. While there exist several accounts of specific cases where the Dempster–Shafer combination rule (2) can be interpreted within PT [46], I rather explore how Bayes’s rule can be understood within ET.

In its basic version, PT implies—among else—the following assumptions:

(i) All possibilities are singletons, in which case $\forall \{A_{i}\}$ and $\forall \{B_{j}\}$ it is either $\left\{A_{i}\right\}\cap \left\{B_{j}\right\}\equiv$ $\{A_{i}\}\equiv$ $\left\{B_{j}\right\}$ or $\left\{A_{i}\right\}\cap \left\{B_{j}\right\}=\emptyset$. In other words, possibilities are not sufficiently nuanced to enable partial overlap. Since it is not possible to generate possibilities beyond those that are included in the incoming bodies of evidence, no novel $C_{k}$ can be generated by Equation (2).
(ii) Although novel possibilities can present themselves, no belief can be allocated to the fear that this may happen. Thus, m(∅)=0. Moreover, the problem of insufficient sample size is effectively dealt with by the Principle of Sufficient Reason. Thus, m(Θ)=0 and the bodies of evidence to be combined take the form $p(\left\{A_{1}\right\}),$ $p(\left\{A_{2}\right\}),\ldots$ $p(\left\{A_{N_{A}}\right\})$ and $p(\left\{B_{1}\right\}),$ $p(\left\{B_{2}\right\}),\ldots$ $p(\left\{B_{N_{B}}\right\})$, respectively, where $N_{A},N_{B}\in N$. Probabilities *p* are subject to the usual constraints $\sum_{i}p\left(\left\{A_{i}\right\}\right)=1$ and $\sum_{j}p\left(\left\{B_{j}\right\}\right)=1$.

In this special case, the Dempster–Shafer combination rule (Equation (2)) boils down to Bayes’ Theorem (see Appendix C for details). However, PT has been greatly extended beyond assumptions (i) and (ii). In particular, *imprecise probabilities* can be defined over an interval $\left[p_{*},p^{*}\right]$ where $p_{*}$ and $p^{*}$ are called *lower probability* and *upper probability*, respectively. With imprecise probabilities, empirical measurement is expected to elicit that p∈ $\left[p_{*},p^{*}\right]$ rather than assessing the exact value of *p*. Correspondingly, two probability distributions are computed, one for $p_{*}$, one for $p^{*}$ [47]. These distributions delimit a *p-box* wherein a set of single-valued distributions can exist [24,48].

Imprecise probabilities are not additive, for $\sum_{i}p_{*}\left(\left\{A_{i}\right\}\right)\leq 1$ and $\sum_{i}p^{*}\left(\left\{A_{i}\right\}\right)\geq 1$. However, $p^{*}\left(\left\{A_{i}\right\}\right)=1-p_{*}\left(\left\{{\bar{A}}_{i}\right\}\right),\forall i$. Assumption (i) does not change if probabilities are imprecise, but (ii) does:

(i)’ ≡ (i)
(ii)’ Although novel possibilities can present themselves, no belief can be allocated to the fear that this may happen. Thus, m(∅)>0. However, it is generally m(Θ)≥0, with strict inequality if at least one probability is lower than $p^{*}$. The bodies of evidence to be combined take the form $\{[p_{*}\left(\left\{A_{1}\right\}\right),$ $p^{*}\left(\left\{A_{1}\right\}\right)],$ $[p_{*}\left(\left\{A_{2}\right\}\right),$ $p^{*}\left(\left\{A_{2}\right\}\right)]\ldots$ $[p_{*}\left(\left\{A_{N_{A}}\right\}\right),$ $p^{*}\left(\left\{A_{N_{A}}\right\}\right)]\}$ and $\{[p_{*}\left(\left\{B_{1}\right\}\right),$ $p^{*}\left(\left\{B_{1}\right\}\right)],$ $[p_{*}\left(\left\{B_{2}\right\}\right),$ $p^{*}\left(\left\{B_{2}\right\}\right)],\ldots$ $[p_{*}\left(\left\{B_{N_{B}}\right\}\right),$ $p^{*}\left(\left\{B_{N_{B}}\right\}\right)]\}$, respectively, where $N_{A},N_{B}\in N$.

Imprecise probabilities can be used to combine traditional probabilistic uncertainty with the uncertainty deriving from relying on too small a sample—the known unknowns. Suppose, for instance, that you are playing for the first time with a die that you suspect may not be fair. Lack of information may prudentially suggest p∈[1/7,1/5] rather than p=1/6. Later on, by throwing the die again and again this interval shrinks down towards the true, precise probabilities. Unless the die is so unfair that the probability of some face(s) is smaller than 1/7 and that of some other face(s) is larger than 1/5, the initial assumption was not incorrect.

When imprecise probabilities are employed in order to deal with known unknowns, upper probabilities are sometimes neglected. The remaining lower probabilities are eventually called *sub-additive probabilities*, to which the standard probability calculus applies [49,50]. In particular, a body of evidence $\{p_{*}\left(\left\{A_{1}\right\}\right),$ $p_{*}\left(\left\{A_{2}\right\}\right),$*…*$p_{*}\left(\left\{A_{N_{A}}\right\}\right)\}$ can be conditioned on $\{p_{*}\left(\left\{B_{1}\right\}\right),$ $p_{*}\left(\left\{B_{2}\right\}\right),$*…*$p_{*}\left(\left\{B_{N_{B}}\right\}\right)\}$ by means of Bayes’s rule [51].

More in general, imprecise probabilities on singletons can be handled just like precise probabilities on partially overlapping sets [52,53]. In order to grasp the rationale for this transformation, suppose that you are dealing with an unfair die where face 1 shows up more often than 1/6 because some lead has been injected just below face 6. Thus, faces 2, 3, 4 and 5 show up less often and face 6 least often. You can understand it as if a portion of the events “face 2” to “face 5,” and a substantial portion of the event “face 6,” have been turned into the event “face 1.” For instance, you should have observed face 2, but you observe face 1 in fact.

**Figure 1:**
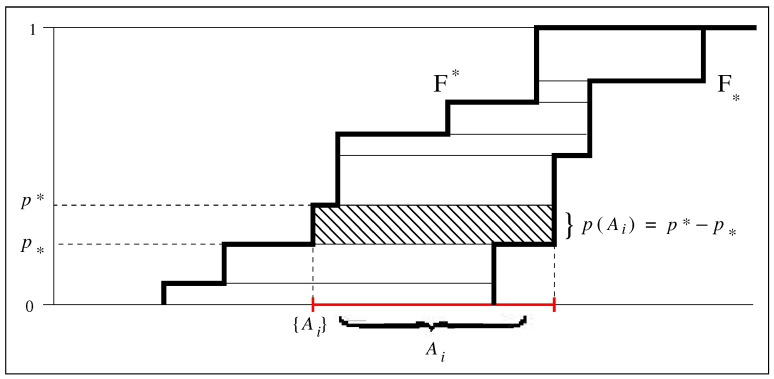
Transformation of imprecise probabilities defined on singletons into single-valued probabilities defined on intervals. Step-wise cumulative lower probability function $F_{*}$ and cumulative upper probability function $F^{*}$ identify intervals $A_{i}$ with probability $p\left(A_{i}\right)=p^{*}-p_{*}$ (the red interval on physical and technical possibilities corresponds to the probability interval $\left[p_{*},p^{*}\right]$). Notably, for any $\left(i,i^{\prime }\right)$ it may happen that $A_{i}\cap A_{i^{\prime }}\neq \emptyset$.

Figure 1 illustrates this transformation for 1-dimensional sets. The lower and upper cumulative functions $F_{*}$ and $F^{*}$ delimit a probability interval $\left[p_{*},p^{*}\right]$ for the singleton $\left\{A_{i}\right\}$. This is the standard format for imprecise probabilities. However, it can also be expressed in terms of a possibility set $A_{i}$ and a single-valued probability $p\left(A_{i}\right)=p^{*}-p_{*}$.

The transformation illustrated in Figure 1 has practical significance. Consider insurance companies facing the problem of evaluating the cost of adverse events without reliable samples, which is by the way the first instance of known unknowns ever identified in economics [54]. For instance, climate change favors wildfires, hence the probabilities that had been measured decades ago no longer apply. This uncertainty concerns a known possibility, namely wildfires, but its probability is unknown—it is a known unknown, indeed. The theory of imprecise probabilities suggests to use probability intervals, which is theoretically sound but offers no guidance as to how the extremes of these intervals might be computed. However, the transformation illustrated in Figure 1 suggests that one may rather attempt to look into technical differences between the climate change-induced wildfires with respect to the purely natural ones, for instance in terms of the length of the dry season, firefighters’ equipment, the strength and direction of winds in specific areas, or else. These features correspond to a set of possibilities that partially overlaps with that of purely natural wildfires by an extent which a technical evaluation can assess.

Note that with the transformation illustrated in Figure 1 we obtain the framework of ET, which is based on sets $A_{i}$ rather than singletons $\left\{A_{i}\right\}$. This transformation is not always one-to-one because of a few special cases when singletons appear along with intervals, but it is one-to-one in most practical applications [52,53].

The duality of singleton-based imprecise probabilities and set-based single-valued probabilities suggests re-formulating assumptions (i)’ and (ii)’ as follows:

(i)” Possibilities are generally represented by sets $A_{1},$ $A_{2}\ldots$ $A_{N_{A}}$, which may intersect with one another. Thus, novel possibilities $C_{k}$ can be generated by Equation (2).
(ii)” Although novel possibilities can present themselves, no belief can be allocated to the fear that this may happen. Thus, m(∅)=0. However, m(Θ)≥0 and the bodies of evidence to be combined take the form $p(A_{1}),$ $p(A_{2}),$*…*$p(A_{N_{A}}),$ $m_{A}\left(\Theta \right)$ and $p(B_{1}),$ $p(B_{2}),$*…*$p(B_{N_{B}}),$ $m_{B}\left(\Theta \right)$, respectively, where $N_{A},N_{B}\in N$.

With assumptions (i)” and (ii)”, we are still within PT, but bodies of evidence must be combined by means of the Dempster–Shafer rule (Equation (2)) instead of Bayes’s Theorem. The main differences with ET are that (a) Probabilities *p* appear instead of masses *m*, and (b) The possibility that m(∅)>0 is ignored.

One remarkable conclusion is that Dempster–Shafer’s combination rule, as well as belief and plausibility functions defined on the $C_{k}$ induced by Equation (2), are well within (an extended version of) PT. Indeed, Arthur Dempster moved initially from imprecise probabilities when he proposed Equation (2) [19]. In the end, the framework of a judge or detective looking for cues instead of a gambler playing dice makes the difference between ET and PT, not the maths.

### 3.2. Evidence Theory and Information Theory

IT [55] assumes that a source emits characters drawn from a known alphabet $A=\{\left\{A_{1}\right\},$ $\{A_{2}\},$ $\ldots \{A_{N}\}\}$. These characters must travel through a noisy channel in order to be communicated to a receiver who is aware that the characters have been drawn from *A*. Noise is able to alter characters. Thus, in order to minimize errors each character $\left\{A_{i}\right\}$ is coded into a set of characters $A_{i}$, with $\|A_{i}\|\;>\;1$ where $\|A_{i}\|$ is the length of the sequence of characters into which each original character is coded. Since noise is unlikely to alter sufficiently many characters of $A_{i}$ to make it unrecognizable, the receiver is most often able to reconstruct the original character. The greater $\|A_{i}\|$, the greater the ability to correct errors, but also the slower the communication because more characters must pass through the channel.

Shannon’s entropy [55], formally similar to thermodynamic entropy, is maximum when characters are equiprobable. It is an average of the information obtained by receiving one character only. Its rationale is that the more uncertain the receiver is about which character she will receive, the more information she obtains upon receiving it.

In ET, testimonies are transmitted to a judge for evaluation. Thus, the context of ET can be likened to that of a communication channel [12]. This is particularly evident in the one-dimensional example mentioned in Section 2, where the numerator of Equation (2) was explained in terms of serial testimonies whereas the denominator reflected parallel testimonies.

However, one crucial difference is that in IT the successful reconstruction of a signal that passed through a noisy channel yields singletons $A_{i}$ that are absolutely different from one another, whereas in ET a similarly successful reconstruction yields sets $A_{i}$ that might intersect with one another. Thus, while Shannon’s entropy measures *discord* between the characters that have been received (weighted by their probabilities), a corresponding magnitude to be defined in ET should measure discord as well as *non-specificity* of the sets $A_{i}$ caused by their intersections.

The quest for a counterpart of information entropy suitable for ET is a very active research field that has not yet reach a universally accepted functional (see [56,57,58,59,60] for discussions and reviews). Several authors have listed desirable properties that this magnitude should possess [58,61,62,63], but none of the magnitudes that have been proposed satisfies all requirements.

The following recent proposal [63] is indicative of the sort of expressions that have been discussed:

(6) $$H\left(A\right)\;=\;-\sum_{A_{i}\in \Theta }\;\frac{Pl\left(A_{i}\right)\;\;\mathrm{lg}\;Pl\left(A_{i}\right)}{e^{Pl\left(A_{i}\right)-Bel\left(A_{i}\right)}}\;\;+\sum_{A_{i}\in \Theta }\;\left[Pl\left(A_{i}\right)-Bel\left(A_{i}\right)\right]$$

where Equations (4) and (5) have been applied with $H\equiv A_{i}$.

The first term of Equation (6) measures discord and reduces to Shannon’s entropy if $A_{i}\equiv \left\{A_{i}\right\}$, which implies that $Bel(A_{i})=$ $Pl(A_{i})=$ $p(\left\{A_{i}\right\})$. The second term measures non-specificity by means of the difference between plausibility and belief.

More recently, researchers are introducing ET entropy measures that rely on the structural isomorphism between entropy and the Hausdorff fractal dimension [64]. Suppose that an FoD entails a set *A* that in its turn entails singletons: A= $\{\left\{A_{1}\right\},$ $\{a_{2}\},$ …}. Then split *A* again and again, generating increasingly smaller sub-sets. This is a fractal structure where an entropy measure can be defined, that unites non-specificity and discord [65]. This framework has been eventually extended to time-varying evidence [66,67].

In an open world, noise can be a source of meaningful novelties rather than a disturbance to be eliminated—such is the case, for instance, of random mutations for living organisms. If IT is applied to the transmission of information through generations by means of the genetic code, noise—random mutations—may make information entropy decrease, rather than necessarily increasing it [68,69]. Specifically, random mutations may make information entropy decrease if they are sufficiently rare.

For the same reason, in an open world the first term of Equation (6) can either increase, because the number of possibilities increase, or decrease, if those novel possibilities are extremely few. By contrast, the second term of Equation (6) has no counterpart in Shannon’s entropy. The difference between $Pl(A_{i})$ and $Bel(A_{i})$ measures to what extent the available evidence goes beyond $A_{i}$ to support some other possibility. Thus, this term measures the ambiguity of communication codes. This is particularly important for human communication, where novel possibilities can arise out of misunderstandings.

The fractal framework requires a structural uniformity that is unlikely to be realized in an open world. However, future improvements may deal with this difficulty.

## 4. Decision-Making by Seeking Coherence

Albeit utility maximization is the most widely employed model of decision-making, experiments on preference reversal demonstrate that humans do not evaluate utility and probability independently of one another [70,71,72]. This is not a mere bias signaling that the basic model requires corrections and adaptations, but rather an indication that a decision model based on these two magnitudes cannot reflect reality. Apparently, humans do not make their decisions by evaluating two magnitudes, but just one.

In ET, either the belief or plausibility expressed by Equations (4) and (5) are meant to express this magnitude [73,74]. Specific decision-makers and specific contexts may either favor the usage of belief or plausibility, but in either case one single magnitude is used to make a decision. In ET, a decision is made as soon as Sherlock Holmes has tightened and coarsened the FoD until arriving at a coherent interpretation of what looked like messy information. Decision is made by seeking coherence.

Note, incidentally, that understanding human decision as seeking coherence blurs the difference between individual and collective decisions. For instance, Dr. Watson may come up with details that stimulate Sherlock Holmes, and the final decision is made when Watson agrees with Sherlock Holmes.

Henceforth, I shall review one basic model of coherence-based decision-making that has been developed independently of ET, namely Constraint Satisfaction Networks (CSNs), two models that include elements of ET, and finally outline the requirements of a decision model that would include all features of ET. Bayesian Networks (BNs) have been added to the list in order to enable comparisons with a better-known tool [75], but they are fundamentally different. BNs describe and prescribe decision-making in atomistic terms, each probability being conditional on the previously computed probability along a directed acyclic graph, or a tree. Coherence is not sought and eventually not achieved, a circumstance that their advocates interpret as positively highlighting undecidable situations [76], whereas researchers advocating CSNs eventually stress that BNs require assuming prior probabilities that are often unknown to decision-makers [77].

CSNs are neural networks whose neurons may represent possibilities, or concepts, or propositions linked to one another by either excitatory or inhibitory connections that represent inferences. Thus, an excitatory connection from neuron A to neuron B means “A implies B” whereas an inhibitory connection means “A implies ¬ B.”

The more and the stronger the excitatory inputs of a neuron, the higher its output; conversely, inhibitory inputs decrease output. The connection between any two nodes *i* and *j* is weighted by a coefficient $w_{ij}$ which is updated at each time step depending to its contribution to neuron output (Hebbian Rule). Updating is reflexive, with $\Delta \;w_{ij}=\Delta \;w_{ji}$. One notable property is that feedbacks between neurons make the network maximize $Consonance=\sum_{i,j}w_{ij}y_{i}y_{j}$.

Consonance maximization means that those neurons are strengthened, that represent possibilities, concepts or propositions that are coherent with one another. Thus, CNSs model decision-making as a search for coherence [78]. Notable applications of CSNs are the elaboration of scientific theories by arranging empirical findings in a network of coherent causal relations, the evaluation of guilt or innocence in a trial by fitting testimonies in a coherent frame, as well as the formation of medical diagnoses out of disparate analyses and symptoms [79,80,81,82,83].

Recently, CSNs have been extended into networks of networks, where the inner networks represent concepts in individuals’ minds that interact in groups or societies [84,85,86,87]. Many of these models differentiate themselves substantially from basic CSNs.

Evidential Networks (ENs) apply Evidence Theory to situations where all evidence is consonant, i.e., there exists sequences such that $A_{i}\subseteq A_{i+1}\subseteq$ $A_{i+2}\ldots$ and such that they include all evidence. These networks have a tree structure (a directed acyclic graph). Since there are no partial intersections between possibilities, Equation (2) reduces to a straightforward extension of Bayes’s Theorem [88,89]. In this respect, ENs are closest to BNs. ENs can be understood as a generalization of BNs to sub-additive probabilities that enable them to overcome—at least to some extent—the objection that prior probabilities are often unknown to decision-makers [77]. However, ENs are meant to minimize m(Θ), which concept is foreign to BNs. In general, applications make hypotheses H coincide with possibilities that include many or all others, hence no human intervention is required to re-formulate hypotheses or coarsen/refine the FoD [90].

Differently from ENs, Valuation Networks (VNs) do exploit the potential of ET in terms of intersecting possibilities [91,92,93,94]. VNs can be represented as a hypergraph whose hyperedges correspond to the possibilities envisaged in the FoD. Intersections between possibilities correspond to common faces between hyperedges; for instance, possibilities $A_{i}=\left\{\alpha ,\beta ,\gamma \right\}$ and $A_{j}=\left\{\beta ,\gamma ,\delta \right\}$ are triangles that have in common the edge $C_{k}=\left\{\beta ,\gamma \right\}$.

**Figure 2:**
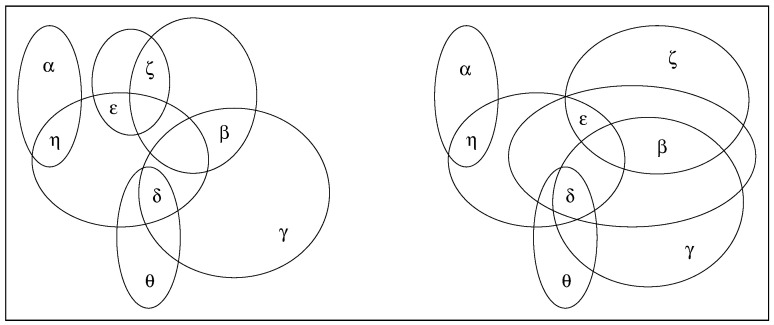
Possibilities arranged as a cyclic hypergraph (**left**) and an acyclic hypergraph (**right**). For each hypergraph, hyperedges rerpresent possibilities whereas their vertices, denoted by Greek letters, represent the elements that they entail; for instance, the possibility $A_{1}=\left\{\alpha ,\eta \right\}$ corresponds to a hyperedge (a segment) of vertices α and η. The cyclic hypergraph on the left is made of hyperedges $A_{1}=\left\{\alpha ,\eta \right\}$, $A_{2}=\left\{\beta ,\zeta \right\}$, $A_{3}=\left\{\delta ,\theta \right\}$, $A_{4}=\left\{\epsilon ,\zeta \right\}$, $A_{5}=\left\{\beta ,\gamma ,\delta \right\}$, $A_{6}=\left\{\delta ,\epsilon ,\eta \right\}$. The acyclic hypergraph on the right can be obtained by removing $A_{2}$ and $A_{4}$ and adding $A_{6}=\left\{\beta ,\delta ,\epsilon \right\}$ and $A_{7}=\left\{\beta ,\epsilon ,\zeta \right\}$. Thus, the acyclic hypergraph has been obtained by coarsening the FoD. Freely redrawn from [92].

While ENs are directed acyclic graphs (trees), VNs are directed acyclic hypergraphs (hypertrees). Figure 2 illustrates the difference between possibilities arranged as a cyclic hypergraph (on the left) and possibilities arranged as an acyclic hypergraph (on the right).

In general, cyclic hypergraphs can be turned into acyclic hypergraphs by coarsening the FoD. For instance, the acyclic hypergraph on the right of Figure 2 can be derived from the cyclic hypergraph on the left by removing $A_{2}=\left\{\beta ,\zeta \right\}$ and $A_{4}=\left\{\epsilon ,\zeta \right\}$ and adding $A_{6}=\left\{\beta ,\delta ,\epsilon \right\}$ and $A_{7}=\left\{\beta ,\epsilon ,\zeta \right\}$ [92].

In VNs, the presence of a substantial m(Θ)>0 may suggest restructuring the FoD. In particular, coarsening can often ease computation with little information waste [36,37]. However, although coarsening can be easily formulated, refinement—which implies envisaging novel possibilities—is not possible in VNs.

Prospectively, let me label Open World Networks (OWNs) a future class of hypergraphs that either because of m(Θ)>0 or m(∅)>0 can either coarsen or refine the FoD. In particular, novel possibilities entering the FoD must interact with the previous ones in ways that cannot be constrained along a pre-determined sequence. Thus, OWNs must be undirected cyclical hypergraphs.

OWNs should avoid that cycling information generates coherence independently of the soundness of the arguments that support it. Separating novel evidence from what has already been used could be a criterion to avoid unproductive cycling [95].

Table 1 compares CSNs, ENs, VNs and OWNs with respect to (a) node output, (b) updating rules, (c) structures, and (d) objective functions.

With the exception of BNs, all other networks maximize or minimize some objective function. CSNs maximize *Consonance*. ENs and VNs minimize m(Θ). OWNs minimize both m(Θ) and m(∅).

CSNs and OWNs have a similar structure (undirected cyclic graphs/hypergraphs), whereas BNs, ENs and VNs are structurally similar to one another (directed acyclic graphs/hypergraphs). ENs and VNs are similar to one another also insofar as they combine evidence by means of Dempster–Shafer’s rule (2), whereas BNs, CSNs and OWNs are quite different from one another in this respect.

## 5. Conclusions

While ET is being increasingly used in data fusion, its ability to deal with non-probabilistic uncertainty has been largely neglected. At the same time, non-probabilistic forms of uncertainty are being increasingly debated in the social sciences, pinning down definitions and differences but without any ability to develop mathematical and computational methods. By connecting these two research areas, I hope to favor the awareness and usage of proper tools.

ET is fascinating because of its unconventional assumptions. In particular, the lack of the complementation operator somehow parallels the dismissal of classical, algorithm-based artificial intelligence as the true model for the human brain by the connectionist revolution of the 1980s. It is an uneasy choice, because instead of providing a ready-made algorithm that (supposedly) reproduces human uncertain reasoning, ET limits itself to offering suggestions for repeated refinement and coarsening of the FoD in the course of an interactive process that interrogates reality, formulates hypotheses, and back again. No ready-made solution, just a broad guidance in the quest for coherence.

Ascribing a positive mass to the void set is even more unconventional an assumption, for which careful mathematical foundations are in need. Intuitively, one may remark that just like m(Θ)>0 is not distributed among the $A_{i}$, the m(∅)>0 is not distributed among anything. In a way, just like m(Θ)>0 hovers above the FoD, the m(∅)>0 hovers above the nihil.

Refusing to separate something like “utility” from something like “probability” is possibly the most striking feature of ET, one that runs against deeply ingrained ideas about what constitutes rationality. Thousands of years before utility maximization, *The Fable of the Fox and the Winegrapes* pointed to the stupidity of the fox who, upon evaluating the probability to reach the grapes to be about zero, updated her utility by convincing herself that the grapes were sour. However, just like a Buddhist *koan*, this fable may hide deeper levels of understanding; one may notice, for instance, that the animal who was unable to separate utility from probability was not a donkey, but a fox. Perhaps, that animal was not so stupid.

## Figures and Tables

**Table 1:** Differences and similarities between BNs, CSNs, ENs, VNs and OWNs with respect to (a) the output generated by single nodes, and its consequences in terms of formulating H or coarsening/tightening Θ ($2^{\Theta }$ denotes the set of all subsets of Θ); (b) updating rules; (c) the structure of the network, and (d) objective functions to be maximized or minimized.

	(a)	(b)	(c)	(d)
	Output	Update	Structure	Objectives
BN	Posterior Conditional Probabilities	Bayes’ Theorem	Directed Acyclic Graph	—
CSN	weighted Σ + excitatory − inhibitory	Hebbian Rule	Undirected Cyclic Graph	Consonance
EN	$H^{A}\equiv A_{i}⊇\forall A_{j}$ $H^{B}\equiv B_{h}⊇\forall B_{k}$	Dempster Shafer	Directed Acyclic Graph	m(Θ)
VN	$\forall \;H\in 2^{\Theta }$ coarsening Θ	Dempster Shafer	Directed Acyclic Hypergraph	m(Θ)
OWN	$\forall \;H\in 2^{\Theta }$ coarsening Θ tightening Θ	Smets	Undirected Cyclic Hypergraph	m(∅), m(Θ)

## Appendix A. Open Worlds

The assumption that novel events might appear, that are currently not in the FoD, is eventually known as *open world*. By contrast, the expression *closed world* means that all possibilities are known in advance.

Shafer’s seminal work on ET did not explicitly discuss this issue, albeit referencing to judges or detectives rather than gamblers implies an open world [10]. The TBM assumes an open world which, after some aggregation, is reduced to a closed world regulated by probability distributions.

The open world assumption is closely tied to rejecting the assumption of a σ-algebra with respect to complementation. Unfortunately, algebras are never mentioned in Shafer’s seminal work [10]. However, it is clear that the complementation operator is not available for incoming evidence, whereas it is available for the hypotheses that the judge or detective conceives. Moving from this observation, the Theory of Hints [27] assumes that although the FoD is not a σ-algebra, hypotheses H are formulated within a subset Ω⊂Θ which is a σ-algebra. I followed this interpretation. By contrast, TBM defines the FoD as a Boolean algebra [13]. There are differences between σ-algebras, which are employed in measure theory, and Boolean algebras, which are mainly employed in artificial intelligence, but both admit the complementation operator. Thus, with either a σ-algebra or a Boolean algebra the possibility set is exhaustive.

In spite of having assumed that the FoD is a Boolean algebra, TBM has been first to formalize the uncertainty that something may happen, that one is not even able to figure out by means of m(∅)>0 [17,18]. This is a clear departure from basic ET, where by definition m(∅)=0 and m(Θ)=1 [10]. The rationale for this formalization is that m(∅)>0 signals high conflict, hence one suspects that something else may happen. The subsequent literature on open worlds within ET has mainly kept the assumptions of TBM [23,96,97,98], which we did as well.

In the earliest version of ET [10], *weights of evidence* are empirical assessments $w(A_{i}):$ $A_{i}\to \left[0,+\infty \right]\;\forall A_{i}$ that feed belief masses *m* as follows:

$$m\left(A_{i}\right)=1-e^{w(A_{i})}\;$$

Thus, with growing evidence the m(.)s grow while m(Θ) decreases. I interpreted m(Θ) as expressing uncertainty over known unknowns and, since I want to have a measure of uncertainty about unknown unknowns as well, I included both m(Θ) and m() in the normalization Equation (1).

The earliest version of ET called a body of evidence $\{m\left(A_{1}\right),$ $m(A_{2}),\ldots \}$ a *basic probability assignment* [10]. This terminology induced much of the subsequent literature to understand ET as an extension of PT, assimilating the FoD to a σ-algebra. I rather followed TBM in ignoring “basic probability assignments” altogether, assuming the existence of masses m(.) and defining belief and plausibility functions upon them [32]. According to this interpretation, ET is built on empirical evidence, the FoD on which it impinges, and the beliefs and hypotheses that humans entertain. Probability never enters the picture.

All versions of ET make use of combination rules, but some of them modify Dempster–Shafer’s rule (2). For instance, TBM eliminates the denominator that redistributes conflicting evidence. The appropriateness of the Dempster–Shafer combination rule will be discussed at greater length in Appendix B.

## Appendix B. Zadeh’s Paradox

Zadeh criticized Depster-Shafer’s combination rule by means of the following example [99]:

Suppose that a patient, P, is examined by two doctors, A and B. A’s diagnosis is that P has either meningitis, with probability 0.99, or brain tumor, with probability 0.01. B agrees with A that the probability of brain tumor is 0.01, but believes that it is the probability of concussion rather than meningitis that is 0.99.

Application of Equation (2) leads to the conclusion that Bel(Tumor)=1.0, which is clearly unrealistic. Zadeh’s paradox originates from mutually exclusive possibilities in a closed world, and most reviews failed to realize that it would generate equally paradoxical results if Bayes’ Theorem were applied without adding the possibility that either doctor is wrong [21,23].

Thus, Zadeh’s paradox induced the conviction that Dempster–Shafer’s combination rule was wrong and sparked a search for alternatives. I grouped these and other alternatives in three categories that are illustrated the ensuing Appendix B.1, Appendix B.2 and Appendix B.1, respectively.

### Appendix B.1. Redistributing Conflict

Since Zadeh’s paradox derives from conflicting evidence, alternative combination rules have been devised that redistribute it among non-conflicting possibilities more efficiently. The list of such alternative rules is quite long [100,101,102].

Here below I report the PCR5 rule, which is generally appreciated for the sensible results that it generates [103,104]. Further improvements are able to accept several bodies of evidence at a time, as well as weighting by reliability and importance [105,106].

$$m_{PCR5}\left(C_{k}\right)\;\;=\;\sum_{A_{i}\cap B_{j}}\;m_{A}\left(A_{i}\right)\;m_{B}\left(B_{j}\right)\;\;+\;\sum_{X\cap C_{k}=\emptyset }\;\left[\;\frac{m_{A}^{2}\left(C_{k}\right)\;m_{B}\left(X\right)}{m_{A}\left(C_{k}\right)+m_{B}\left(X\right)}\;\frac{m_{B}^{2}\left(C_{k}\right)\;m_{A}\left(X\right)}{m_{B}\left(C_{k}\right)+m_{A}\left(X\right)}\;\right]\;$$

where $X\in 2^{\Theta }$.

### Appendix B.2. Channelling Conflict Elsewhere

There are several alternatives to redistributing conflicting evidence among non-conflicting possibilities. Alternatives include transfering conflicting evidence to the FoD, transfering it to the union of conflicting possibilities, as well as transfering it to ∅ as in TBM [23].

If Equation (3) is applied to Zadeh’s example, it delivers m(∅)=0.9999. In other words, by listening to two experts saying opposite things one concludes that the truth might possibly lie somewhere else.

### Appendix B.3. Reframe the Problem

One even more radical alternative builds on Shafer’s own suggestion that the FoD can be refined and further refined until one finds pieces of evidence that are sufficiently detailed to be independent of one another [38]. In the case of Zadeh’s paradox, the method would consist of reframing the diagnoses of the two doctors until uncovering details that highlight areas of overlap.

For instance, Haenni remarked that diseases may not come alone [21]. Suppose that doctor A rather expresses a probability 0.99 for meningitis either alone, or in conjunction with either tumor or concussion, or both. With a similar re-interpretation of doctor B’s diagnosis, Dempster–Shafer’s rule yields sensible outcomes [21]. Likewise, Boivin solved the puzzle by reasoning that the patient may compute the union of the two diagnoses, obtaining similar conclusions [22].

Refining the FoD does not contradict the policy of moving conflicting evidence into m(∅)>0. Rather, m(∅)>0 signals that some coarsening or refinement of the FoD is in order.

## Appendix C. From Dempster–Shafer to Bayes’ Theorem

In this appendix I show that, under assumptions (i) and (ii), the Dempster–Shafer combination rule (2) reduces to Bayes’ Theorem. Let us also assume that hypotheses are automatically generated by the combination of evidence through Equation (2), hence Equations (4) and (5) are not necessary. With these assumptions, beliefs coincide with probabilities and, because of condition (i), beliefs at previous points in time can be expressed in terms of prior evidence $\{p\left(\left\{A_{1}\right\}\right),$ $p(\left\{A_{2}\right\}),\ldots$ $p(\left\{A_{N_{A}}\right\})\}$. Thus, updating beliefs means conditioning posterior probabilities on prior probabilities:

Prior Probability: $Bel^{t}\left(H_{i}\right)\equiv p\left(\left\{A_{i}\right\}\right)$, ∀i
Posterior Probability: $Bel^{t+1}\left(H_{i}\right)\equiv p\left(\left\{A_{i}\right\}|\left\{B_{j}\right\}\right)$, ∀i,j

For simplicity, and without loss of generality, let us assume that $\exists p,q:\left\{A_{p}\right\}\equiv \left\{B_{q}\right\}$ whereas ∀i,j≠{p,q} it is $\left\{A_{i}\right\}\cap \left\{B_{j}\right\}=\emptyset$. Let us feed Equation (2) into $Bel^{t+1}\left(H_{p}\right)$ while highlighting the time stamp by means of a superscript:

$$\begin{array}{ccccc} p(\left\{A_{p}^{t}\right\}|\left\{B_{q}^{t+1}\right\}) & \overset{\left(a\right)}{=} & \frac{p\left(\left\{A_{p}^{t}\right\}\right)\;p\left(\left\{B_{q}^{t+1}\right\}\right)}{1\;-\;\sum_{\begin{array}{c} i\neq p\\ j\neq q \end{array}}\;p\left(\left\{A_{i}^{t}\right\}\right)\;p\left(\left\{B_{j}^{t+1}\right\}\right)} & \overset{\left(b\right)}{=} & \frac{p\left(\left\{B_{q}^{t}\right\}\right)\;p\left(\left\{A_{p}^{t+1}\right\}\right)}{p(\left\{B_{q}^{t+1}\right\})}\\ & \overset{\left(c\right)}{=} & \frac{p\left(\left\{B_{q}^{t}\right\}|\left\{A_{p}^{t+1}\right\}\right)\;p\left(\left\{A_{p}^{t+1}\right\}\right)}{p(\left\{B_{q}^{t+1}\right\})} & \end{array}$$

which is Bayes’ Theorem for $\left\{A_{p}\right\}$ and $\left\{B_{q}\right\}$.

Passage (a) is a straightforward application of Equation (2). The denominator of passage (b) is obtained by remarking that albeit $\left\{A_{p}^{t}\right\}$ and $\left\{B_{q}^{t+1}\right\}$ overlap, $\left\{B_{q}^{t+1}\right\}$ comes at a later point in time. The numerator of passage (b), as well as passage (c), requires a time inversion of the arrival of bodies of evidence $\left\{p\left(\left\{A_{1}\right\}\right),p\left(\left\{A_{2}\right\}\right),\ldots p\left(\left\{A_{N_{A}}\right\}\right)\right\}$ and $\left\{p\left(\left\{B_{1}\right\}\right),p\left(\left\{B_{2}\right\}\right),\ldots p\left(\left\{B_{N_{B}}\right\}\right)\right\}$, respectively. This is possible because the sequence of arrival has no impact on the Dempster–Shafer rule, and it is the very same logic employed in the standard demonstration of Bayes’ Theorem.
